# Preoperative integrated oxidative stress score as a prognostic factor for predicting clinical outcomes in breast cancer patients received neoadjuvant chemotherapy: a real-world retrospective study

**DOI:** 10.7150/ijms.109915

**Published:** 2025-02-26

**Authors:** Li Chen, Caixia Dai, Shu Peng, Hao Dong, Xiangyu Wang, Yanfei Liu, Jing Wang

**Affiliations:** 1 Department of Breast Surgical Oncology, National Cancer Center/National Clinical Research Center for Cancer/Cancer Hospital, Chinese Academy of Medical Sciences and Peking Union Medical College, Beijing, 100021, P.R. China.; 2 Department of Hepatobiliary&Pancreatic Surgery, Zhongnan Hospital of Wuhan University, Wuhan, Hubei, 430071, P.R. China.; 3 Department of Thoracic Surgery, Tongji Hospital, Tongji Medical College, Huazhong University of Science and Technology, Wuhan, Hubei 430030, P.R. China.; 4 Department of Oncology, Puren Hospital Affiliated to Wuhan University of Science and Technology, Wuhan, Hubei 430081, P.R. China.

**Keywords:** breast cancer, breast cancer integrated oxidative stress score, neoadjuvant chemotherapy, nomogram, survival

## Abstract

**Objective:** The current study aims to investigate the prognostic value of breast cancer integrated oxidative stress score (BCIOSS) in patients with breast cancer who received neoadjuvant chemotherapy (NACT).

**Methods:** A retrospective analysis of 104 breast cancer patients who underwent NACT from June 2009 to December 2015 was performed. The differences of BCIOSS of breast cancers in regard to variables were analyzed using Chi-square test and Fisher's exact test. The Kaplan-Meier method was used to evaluate survival curve between low BCIOSS group and high BCIOSS group, and the two groups were compared by Log-rank tests at the individual index level. The univariate and multivariate Cox regression analyses were established by the important predictive factors determined based on univariate analysis. The nomograms were further conducted based on the factors by the multivariate analyses.

**Results:** Patients were assigned to low BCIOSS group (BCIOSS≤2.54) or high BCIOSS group (BCIOSS>2.54) via ROC curve. BCIOSS was a latent prognostic factor for patient survival [DFS; hazard ratio (HR): 0.163, 95%CI: 0.045-0.596, P=0.006; and OS; HR: 0.168, 95%CI: 0.056-0.500, P=0.001]. Patients with a high BCIOSS had longer survival time than those with a low BCIOSS (DFS: χ^2^=7.317, P=0.0068; and OS: χ^2^=9.407, P=0.0022). Calibration curves shown that the predicted line conformed well to the reference line for the 5-year survival category. DCA revealed that the nomograms conducted had a better clinical predictive application than only by BCIOSS.

**Conclusion:** BCIOSS is a latent prognostic factor, and patients with high oxidative stress scores have a better prognosis and longer survival time.

## Introduction

Breast cancer is the most common type of malignant tumors in women 1. Data from the International Agency for Research on Cancer (IARC) shows that the morbidity rate of breast cancer ranks second and the mortality rate ranks fourth in the world. Compared with other malignant tumors (hepatoma, carcinoma of the lungs, and pancreatic cancer), the prognosis and survival outcomes of breast cancer are relatively satisfactory. However, advanced and distant metastatic breast cancer has poor prognosis because of its occurrence and metastasis 2, 3. Because of the strong heterogeneity of tumors, patients with equal immunohistochemical subtypes and tumor-node-metastasis (TNM) stages are significantly different 4, 5. That is to say, although there are known risk factors, such as immunohistochemical subtype and TNM stage system, some subtle indicators still affect the prognosis of breast cancer patients.

Reactive oxygen species (ROS), a class of molecules with high biological activity, are supposed to be normal residual products of many cellular processes 6. ROS play a crucial role in cell metabolism: 1) ROS, as a signal transducer, activate angiogenesis, cell proliferation, migration, and invasion at low to medium levels; 2) ROS destroy nucleic acids, proteins, membranes, and lipids, resulting in high levels of cell death 7. ROS play a noteworthy role in the body's defense and killing of tumor cells in many malignant tumors 8, 9. Oxidative stress is a state of linkage disequilibration between oxidants and antioxidants and is associated with the development, occurrence, and aggressiveness of tumors 10, 11. Research has shown that high oxidative stress increases the risk of developing tumors due to impaired antioxidant systems; ROS in oxidative stress will lead to post-translational modification and genetic instability of cancer-involved proteins 12-14.

A large amount of studies declared that ROS played a critical role in tumor tissues. One study on critically ill polytrauma patients with multiple injuries shown significant statistical differences in interleukin 6, total protein, serum albumin, lactate dehydrogenase, and C-reactive protein (CRP) levels with antioxidant treatment compared with those without antioxidant treatment 15. Another study indicated that inflammation and oxidative stress might play important roles in inducing multiorgan damage in a mouse model, and sleep-deprived mice had significantly higher levels of total bilirubin (TBIL), lactic dehydrogenase (LDH), blood urea nitrogen (BUN), creatine phosphokinase myocardial band (CKMB), and glutamic pyruvic transaminase (GPT) 16. Another study also shown that they conducted a systematic oxidative stress scoring system to calculate the prognosis of colorectal carcinoma patients according to oxidative stress indexes 17. These results suggested that biochemical markers might be effective indicators of systemic oxidative stress.

At present, assessment of systemic oxidative stress, antioxidants, and prognosis is crucial for the preventive and therapeutic effects on tumors, such as colorectal carcinoma and T lymphoblastic lymphoma/leukemia 17, 18. However, the relation between systemic oxidative stress and the prognosis of breast cancer patients is still unclear. In the current study, we aim to explore the potential prognostic value of systemic oxidative stress in terms of biochemical markers of oxidative stress. We conduct an integrated oxidative stress score, named breast cancer integrated oxidative stress score (BCIOSS), and investigate the potential prognostic significance of BCIOSS in breast cancer patients who underwent NACT.

## Materials and Methods

### Patients and study design

A total of 104 female patients diagnosed breast cancer underwent NACT between June 2009 and December 2015 at our hospital were enrolled into this study. We searched and collected clinical data, pathological data, and follow-up data from electronic medical records. This study was approved by the ethics review committee of the Cancer Hospital, Chinese Academy of Medical Sciences. And this study was performed in compliance with the 1964 Declaration of Helsinki and its later amendments. The patients were selected and signed informed consent forms.

### Inclusion criteria and exclusion criteria

The inclusion criteria were as follows: 1) diagnosis of breast cancer by histopathology; 2) all enrolled patients received surgical operation after NACT; 3) without anti-tumor therapy before enrollment in our hospital; 4) complete clinical pathology data and follow-up information; and 5) absence of hematological diseases, inflammation, or infection. Patients were excluded if they 1) had multiple primary malignant tumors; 2) lacked definite and clear diagnosis and treatment information; 3) had chronic inflammatory or autoimmune diseases; 4) had cardiovascular disease, kidney dysfunction, and metabolic diseases; and 5) received blood transfusion treatment.

### Breast cancer integrated oxidative stress score (BCIOSS)

In the current study, the BCIOSS was composed of BUN, albumin (ALB), direct bilirubin (DBIL). BCIOSS was calculated as below: 0.074 × ALB (g/L) - 0.094 × DBIL (μmol/L) - 0.099 × BUN (mmol/L), referred to a previous study 17. These blood indicators were detected on the first day after admission in patients with breast cancer.

### Follow-up

Follow-up data were collected via routine outpatient, inpatient, telephonic interviews. The follow-up schedule was based on the NCCN guidelines. In the current study, disease free survival (DFS), which was calculated from the time from operation to distant disease progression and metastasis, local recurrence of the tumor. Overall survival (OS) was calculated as the time from operation to death or last follow-up.

### Statistical analysis

SPSS statistics software (version 23.0), GraphPad Prism software (version 8.0), and R (version 4.2.2; URL: http://www.R-project.org/) were used for all statistical analyses. The optimal cutoff value for BCIOSS was calculated by ROC curve. The variables of breast cancer by BCIOSS were analyzed using Chi-square test and Fisher's exact test. The Kaplan-Meier method was used to evaluate survival curve between low BCIOSS group and high BCIOSS group, and the two groups were compared by Log-rank tests at the individual index level. The univariate Cox proportional hazard model was performed the enrolled variables, and the multivariate Cox regression analyses were established by the important predictive factors determined based on univariate analysis. The nomograms were further conducted based on the factors by the multivariate analyses. The accuracy of the predictive performance was assessed by comparing the observation results and prediction results using calibration curve and decision curve analysis (DCA). P<0.05 was supposed to indicate a statistically significant result.

## Results

### Study population and the characteristics

There were 104 breast cancer patients enrolled the study. The optimal BCIOSS cutoff value was determined to be 2.54. Then, the patients were separated into two groups: low BCIOSS group (BCIOSS≤2.54) and high BCIOSS group (BCIOSS>2.54). Compared to patient characteristics, BCIOSS was associated with ultrasonic sound-breast imaging reporting and data system (US-BI-RADS) (P=0.044) and mammography-lymph node metastasis (M-LNM) (P=0.015) (**Table 1**).

### The common hematologic index and oxidative stress indicators

We used the median values for these hematologic indices and oxidative stress indicators as the cut-off values. **Table 2** shown the distribution of common hematologic indices and systematic oxidative stress indices in the patients. Based on the hematologic index, BCIOSS was significantly related to homocysteine (HCY), red blood cells (R), and eosinophils (E) (P<0.05). Of all enrolled oxidative stress indicators, BCIOSS was found to be significantly related to albumin and direct bilirubin (P < 0.05).

### Association between BCIOSS and chemotherapy

In this study, there were 74 cases received postoperative chemotherapy after NACT and surgery. After two cycles of NACT, we evaluated the response to breast cancer, and all patients who received NACT were effectively relieved, except for one patient who had progressive disease. The toxic side effects of chemotherapy, including NACT and postoperative chemotherapy, were mainly gastrointestinal and hematologic reactions. After receiving chemotherapy when received surgery, there was no statistically significant difference between the two groups (P > 0.05, **Table 3**).

### The relationship between BCIOSS and molecular pathology

Immunohistochemistry was performed to detect pathological molecular indicators, such as androgen receptor (AR), epidermal growth factor receptor (EGFR), E-cadherin (E-cad), and Topoisomerase II-α (TOP2A). There were no differences in molecular pathology between the two groups (P > 0.05, **Table 4**).

### The univariate and multivariate analyses for DFS and OS

The multivariate Cox regression analysis was applied to determine potential factors that were indicated to be significant in univariate regression analysis. BCIOSS, total bile acid (TBA), carcinoembryonic antigen 153 (CA153), ultrasonic sound-lymph node metastasis (US-LNM), pathological tumor size (P-tumor size), postoperative endocrine therapy were potential prognostic factors for DFS. Furthermore, BCIOSS and postoperative endocrine therapy were potential prognostic factors for OS. Detailed information was presented in **Table 5**.

### Survival analysis by BCIOSS

**Figure 1:**
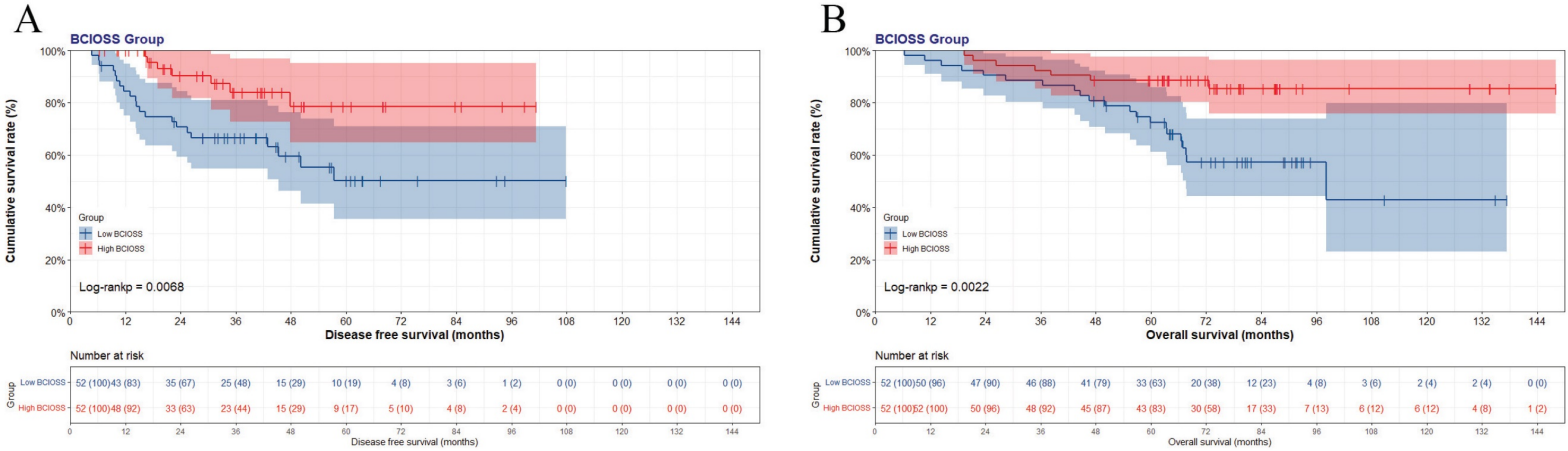
Kaplan-Meier curve of BCIOSS divided group: (A) disease free survival (DFS) and (B) overall survival (OS).

In view of the optimal BCIOSS cut-off value, the mean DFS time was 37.82 months in low BCIOSS group, and 38.59 months in high BCIOSS group. The mean OS time was 66.38 months in low BCIOSS group, and 76.66 months in high BCIOSS group. Patients with high BCIOSS had longer survival time than those with low BCIOSS (χ^2^=7.317, P=0.0068 and χ^2^=9.407, P=0.0022) (**Figure 1**). Moreover, the 1-, 3-, and 5-year survival rates for DFS and OS in low BCIOSS group were 89.4%, 72.2%, 54.5%; and 95.8%, 87.5%, 78.6%, respectively. Furthermore, the 1-, 3-, and 5-year survival rates for DFS and OS in high BCIOSS group were 94.4%, 77.5%, 72.7%; and 100.0%, 92.9%, 82.1%, respectively.

### Nomograms constructed

**Figure 2:**
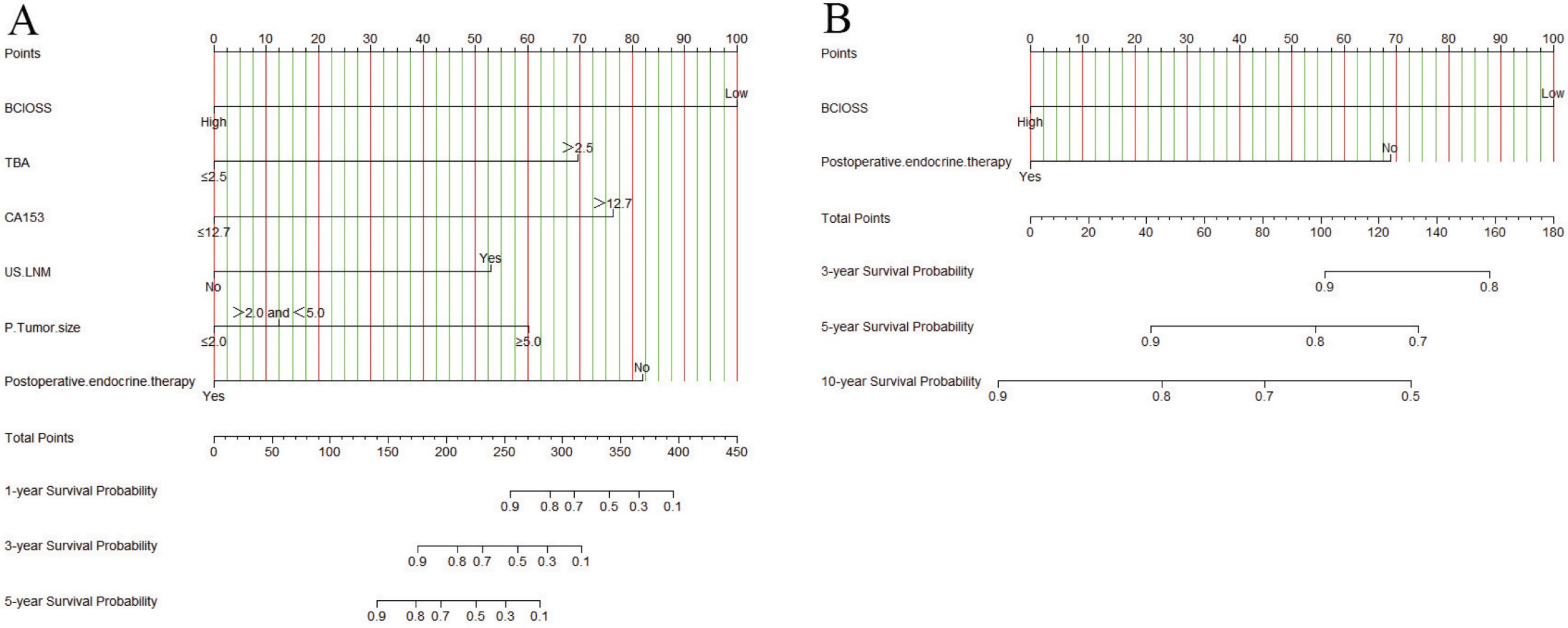
Nomogram based on BCIOSS for predicting disease free survival (A) and overall survival (B).

**Figure 3:**
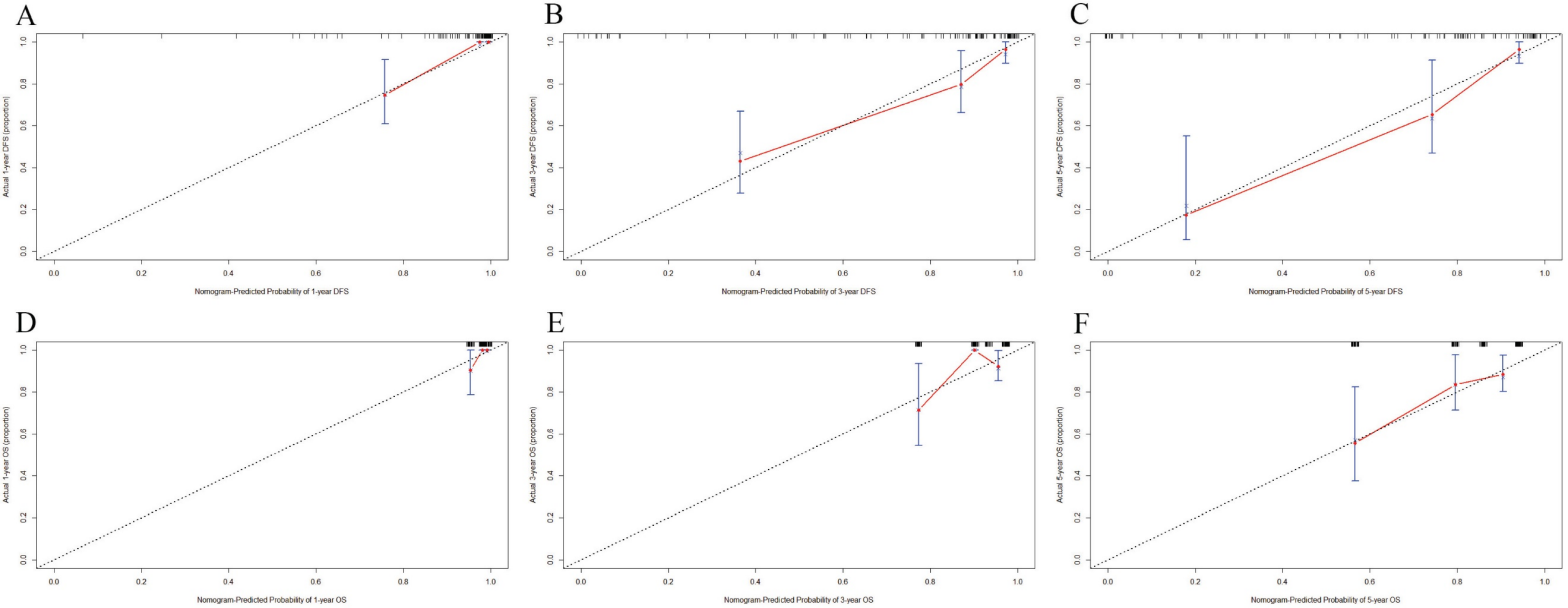
Calibration curves predicting 1-, 3-, 5-year disease free survival (DFS) and overall survival (OS). A) for predicting the 1-year DFS; 2) for predicting the 3-year DFS; C) for predicting the 5-year DFS; D) for predicting the 1-year OS; E) for predicting the 3-year OS; F) for predicting the 5-year OS.

A nomogram for individualized assessment was established using multivariate analysis. According to this nomogram, patients with higher grades had a lower survival probability. Nomogram for DFS included BCIOSS, TBA, CA153, US-LNM, P-tumor size, and postoperative endocrine therapy. Nomograms for OS included the BCIOSS and postoperative endocrine therapy. These nomograms were shown in **Figure 2**. Calibration curves shown that the predicted line conformed well to the reference line for the 5-year survival category (**Figure 3**).

### Predictive accuracy by decision curve analysis (DCA) and time dependent ROC for DFS and OS

**Figure 4:**
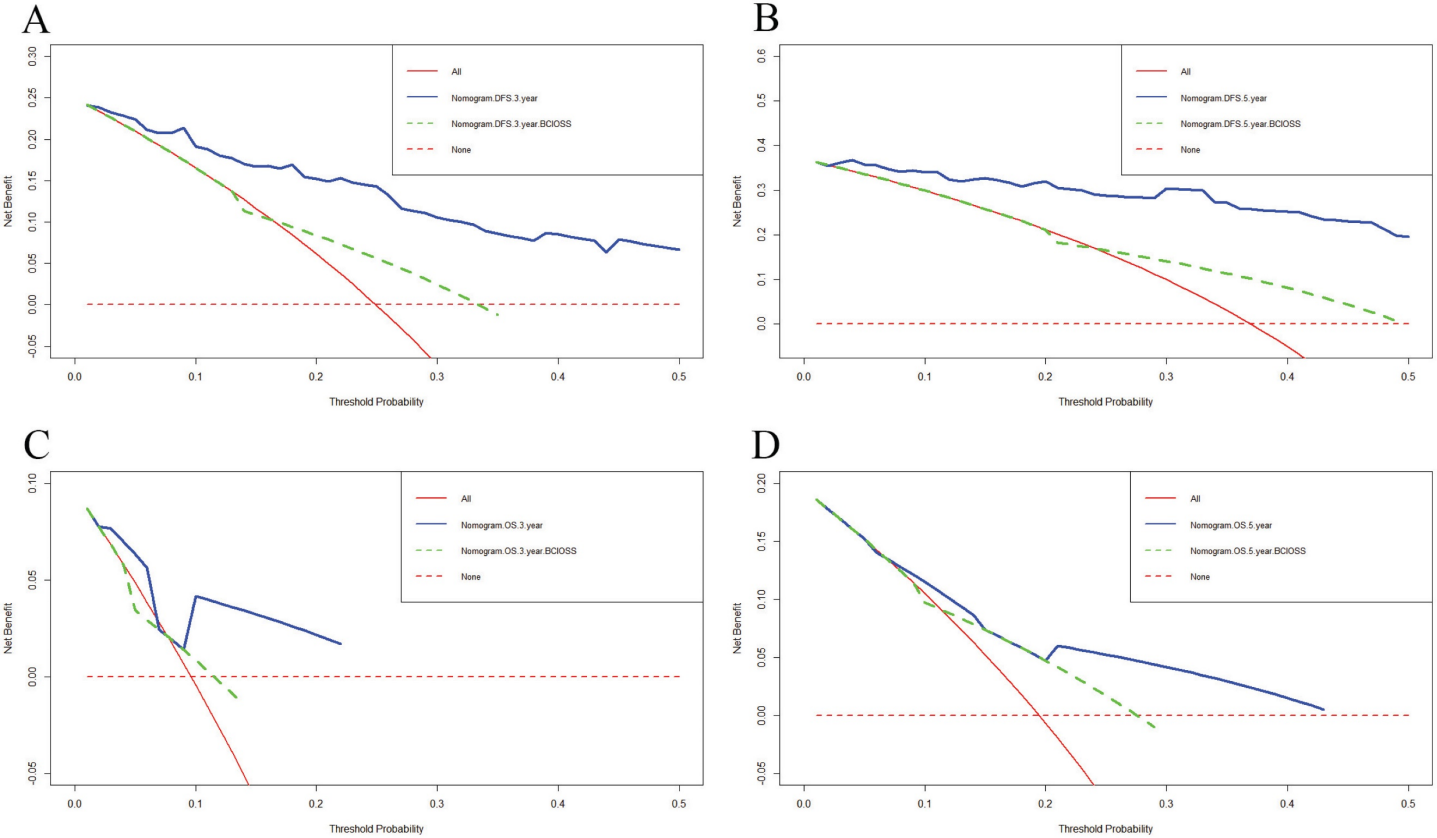
Decision curve analysis (DCA) for evaluating predictive value of the nomogram and the BCIOSS on DFS and OS. A) DCA of the nomogram and BCIOSS for predicting the 3-year DFS; B) DCA of the nomogram and BCIOSS for predicting the 5-year DFS; C) DCA of the nomogram and BCIOSS for predicting the 3-year OS; D) DCA of the nomogram and BCIOSS for predicting the 5-year OS.

**Figure 5:**
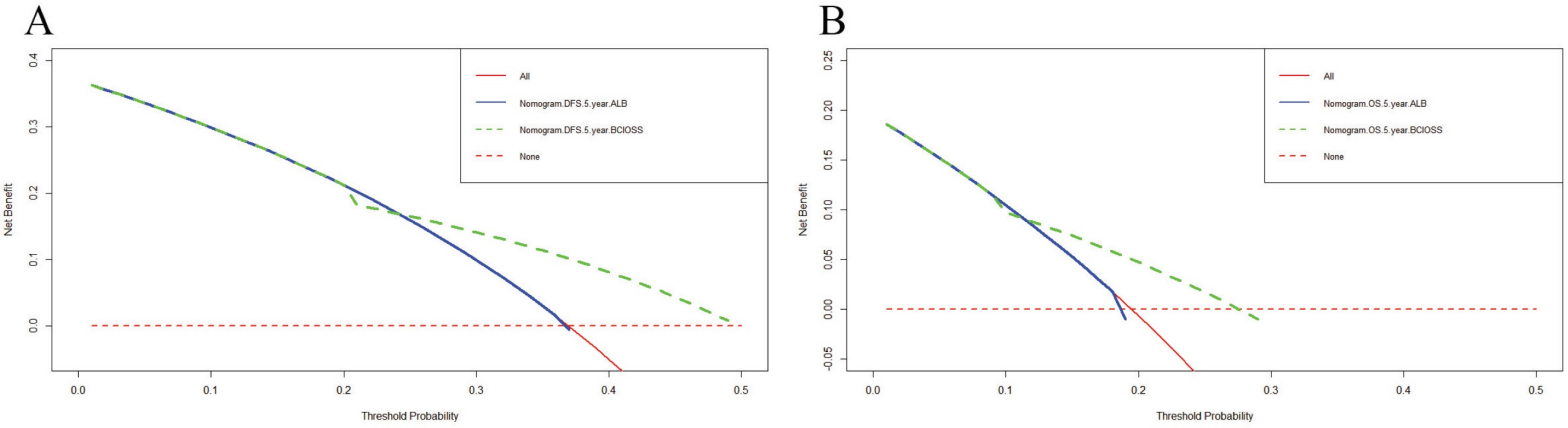
Decision curve analysis (DCA) evaluating BCIOSS and ALB in prediction of 5-year DFS (A) and 5-year OS(B).

DCA was applied to appraise the benefits and clinical utility of different survival time points between the nomogram model and BCIOSS alone. The results shown that the nomogram of the 3-and 5-year survival time had a better predictive value than BCIOSS alone (**Figure 4**). We also analyzed the clinical efficacy of BCIOSS and ALB. Compared with ALB, the BCIOSS had better clinical predictive value by DCA (**Figure 5**).

**Figure 6:**
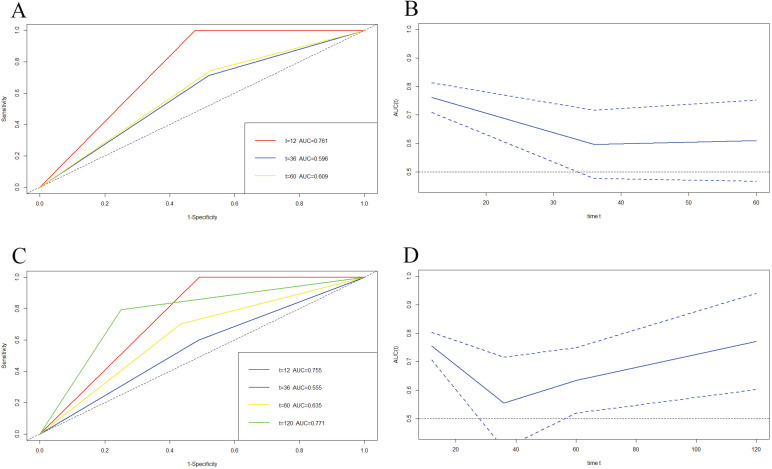
Time-dependent ROC curve for 1-, 3-, 5-year or 10-year survival. A) Time-dependent survival ROC curve for 1-, 3- and 5-year DFS survival; B) AUC and 95%CI changes over 1-, 3-, and 5-year DFS times; C) Time-dependent survival ROC curve for 1-, 3-, 5-, and 10-year OS times; D) AUC and 95%CI changes over 1-, 3-, 5-, and 10-year OS times.

We also used time-dependent ROC (TDROC) and AUC analyses to appraise the prediction accuracy of DFS and OS. TDROC curve of BCIOSS was used to predict 1-, 3-, 5-year or 10-year survival rates. The time-dependent ROC analysis shown that the prognostic accuracy of BCIOSS were 0.761 at 1 year, 0.596 at 3 year, 0.609 at 5 year for DFS (**Figure 6A**), and **Figure 6B** shown the AUC and 95%CI changes over 1-, 3-, and 5-year DFS survival rate. In addition, TDROC analysis shown that the prognostic accuracy of BCIOSS were 0.755 at 1 year, 0.555 at 3 year, 0.635 at 5 year, 0.771 at 10 year for OS, respectively (**Figure 6C**), and **Figure 6D** shown the AUC and 95%CI changes over 1-, 3-, 5-, and 10-year OS survival rate.

## Discussion

Oxidative stress is closely related to formation, advancement, and prognosis of malignant tumors 19, 20. Prior to tumor determination, superfluous oxidants can cause DNA damage and increase the incidence of tumors 21. The reduction in oxidation levels induced by antioxidants may diminish the ability to kill cancer cells, thereby leading to the development of cancer and a reduction in therapeutic effects 22. Breast cancer is a complicated disease that involves tumors and stromal cells 23. Carcinoma-associated fibroblasts (CAFs) located in the CAFs in cancer stroma enhance angiogenesis and promote tumor growth in the tumor microenvironment 24. Under systematic oxidative stress, CAFs in the tumor matrix discharge high-energy nutrients to provide fuel for cancer cells, further stimulating cancer cell growth 25. Oxidative stress is a prominent factor in the progression of breast cancer; however, the relationship between prognosis and the level of systemic oxidative stress remains not known.

In the current study, we explored the latent prognostic significance of BCIOSS in breast cancer patients who underwent NACT and developed a prognostic nomogram model including BCIOSS. The BCIOSS was calculated using ALB, DBIL, and BUN levels. Combined with our data, we included most of the reported biochemical markers related to oxidative stress, such as LDH, ALB, CRP, TBIL, DBIL, SOD, and FIB. In Li L's study, serum LDH > 244 U / L before the T-DM1 treatment was prognostic risk factors for patients with advanced HER2 positive breast cancer receiving T-DM1 treatment, and LDH uptrend after T-DM1 treatment was also related to the poor prognosis 26. Grupińska J's study demonstrated that adjuvant chemotherapy causes systemic inflammation, manifested by increased hs-CRP and altered markers of oxidative stress in the blood of breast cancer patients 27. Li Y's study also indicated that significant expression of superoxide dismutase in luminal B breast cancer and its potential as a prospective marker for this specific molecular subtype 28. Another study also shown that albumin-bilirubin (ALBI) score has high prognostic ability for survival time in breast cancer with liver metastasis after surgery 29. We analyzed the prognostic significance of systematic oxidative stress according to biochemical oxidative stress markers and the significant differences in ALB and DBIL levels.

Based on the univariate and multivariate analyses, the potential independent predictors of DFS were mainly associated with BCIOSS, TBA, CA153, US-LNM, P-tumor size, and postoperative endocrine therapy, and the potential independent predictors of OS were mainly associated with BCIOSS and postoperative endocrine therapy. Liu YH's study demonstrated that IOSS is a nonspecific tumor predictor based on available oxidative stress index, and low IOSS is found to be a vigorous factor of better prognosis in stage III gastric cancer 30. In our study, BCIOSS is a potential prognostic indicator, and lower BCIOSS is associated with poorer prognosis and shorter survival time. The 1-, 3-, and 5-year survival rates in high BCIOSS group were higher than those in low BCIOSS group.

We then established a prognostic nomogram identified by BCIOSS and other indicators that could provide higher accuracy in predicting 1-, 3-, 5-year or 10-year survival probabilities than single traditional prognostic indicators. We also used calibration curves to evaluate the probability in patients with breast cancer between prediction and observation, and the results indicated that the predicted line conformed well to the reference line for the 5-year survival category. DCA was used to determine the benefits and clinical utility of different survival time points between the nomogram and BCIOSS, and the results shown that the nomogram of the 3-and 5-year survival probabilities had better predictive clinical application than BCIOSS alone.

Moreover, we went a step further to compare the benefits and clinical utility of BCIOSS and ALB, and the results shown that the nomogram for BCIOSS displayed a better clinical predictive usefulness than ALB. Furthermore, the time dependent ROC curve and AUC were used to evaluate the 1-, 3-, 5-year or 10-year survival rates, and the results indicated that the value of AUC and 95% CI in predicting the 1-year DFS rate and 10-year OS rate were the highest; however, the value of AUC and 95%CI in predicting the 3-year survival rate was lower than other survival time points. These results shown that BCIOSS had a prominence influence on the prognosis of breast cancer and demonstrated the convincingness traditional biomarkers, such as ALB, in improving the prognostic ability of breast cancer patients.

Several reasonable mechanisms expound the relationship between BCIOSS and breast cancer prognosis. BCIOSS is composed of three parts, including the levels of ALB, DBIL, BUN in the peripheral blood. ALB is associated with inflammation, nutritional status, and antioxidant function 31-33. Elevated ALB can prolong survival in different tumors 34-37. Owing to its antioxidant function, bilirubin is thought to be an anticancer factor. However, the relationship between bilirubin levels and tumor prognosis is contradictory. Evidence has shown that increased bilirubin levels have a worse prognosis in rectal, lung, colorectal cancer 38-40. BUN is discharged by the kidney; however, systemic oxidative stress decreases the ability of the kidney to exhaust urea, resulting in an increase in the level of BUN in peripheral blood, which further influences antioxidant treatment 41, 42. Furthermore, the composition of BUN is the main approach to debase ammonia 43. Supposing that the composition of BUN is obstructed, this results in an increase in the level of ammonia, which facilitates the production of reactive oxygen species. Elevated BUN is related to shorter survival time in different tumors, including carcinoma of the lungs and breast cancer 44, 45.

Additionally, several studies have reported that antioxidants could reduce the therapeutic effect of breast cancer and may even be conducive to the progression of breast cancer. Vitamin E, an antioxidant, can remarkably decrease reactive oxygen species and expression of P53, then to promote the cell proliferation of MCF-7 46. Tamoxifen, an important drug for endocrine therapy of breast cancer, can induce apoptosis in MCF-7 cells by inducing an increase in ROS in the mitochondria, but Vitamin C can protect cancer cells from tamoxifen-induced oxidation, thus inhibiting the death of MCF-7 cells 47. These findings indicate a complex relationship between oxidative stress and breast cancer.

However, this clinical study had some limitations. First, this was a retrospective study on breast cancer patients with a relatively small sample size. More patients should be enrolled, and validated the prognostic value of BCIOSS in the further study. Second, selection deviation is difficult to dispel because of the eligibility criteria. Third, the nomogram was determined by restricted independent factors and lacked external validation. Finally, owing to clinical limitations, the specific mechanisms of oxidative stress and the indicators included remain unclear. Therefore, further clinical studies with more patients are required to verify our results.

## Conclusion

In conclusion, BCIOSS is a breast cancer-integrated oxidative stress score that stems from a combination of oxidative stress indicators. BCIOSS can predict the prognosis of breast cancer patients, and high oxidative stress scores are significantly associated with better prognosis and longer survival time. The nomogram, which combines BCIOSS and other characteristics, can be a predictive layering tool for improving clinical decision making.

## Figures and Tables

**Table 1:** Patient characteristics

	Level	Low BCIOSS	High BCIOSS	p
n		52	52	
Age	<46	21 (40.4)	27 (51.9)	0.325
	≥46	31 (59.6)	25 (48.1)	
BMI	<23.63	22 (42.3)	27 (51.9)	0.432
	≥23.63	30 (57.7)	25 (48.1)	
Family history	No	38 (73.1)	42 (80.8)	0.485
	Yes	14 (26.9)	10 (19.2)	
Menarche age	<14	18 (34.6)	21 (40.4)	0.685
	≥14	34 (65.4)	31 (59.6)	
Menopause	No	29 (55.8)	35 (67.3)	0.314
	Yes	23 (44.2)	17 (32.7)	
ABO blood type	A	14 (26.9)	14 (26.9)	0.504
	B	20 (38.5)	14 (26.9)	
	O	13 (25.0)	15 (28.8)	
	AB	5 (9.6)	9 (17.3)	
US-primary tumor site	Upper outer quadrant	30 (57.7)	40 (76.9)	0.130
	Lower outer quadrant	4 (7.7)	4 (7.7)	
	Lower inner quadrant	3 (5.8)	0 (0.0)	
	Upper inner quadrant	11 (21.2)	7 (13.5)	
	Central	4 (7.7)	1 (1.9)	
US-LNM	No	37 (71.2)	32 (61.5)	0.406
	Yes	15 (28.8)	20 (38.5)	
US-BIRADS	4	7 (13.5)	3 (5.8)	0.044
	5	15 (28.8)	27 (51.9)	
	6	30 (57.7)	22 (42.3)	
M-primary tumor site	Upper outer quadrant	32 (61.5)	33 (63.5)	0.795
	Lower outer quadrant	2 (3.8)	4 (7.7)	
	Lower inner quadrant	4 (7.7)	2 (3.8)	
	Upper inner quadrant	10 (19.2)	7 (13.5)	
	Central	1 (1.9)	1 (1.9)	
	Unknown	3 (5.8)	5 (9.6)	
M-LNM	No	39 (75.0)	26 (50.0)	0.015
	Yes	13 (25.0)	26 (50.0)	
M-BIRADS	4	6 (11.5)	6 (11.5)	0.451
	5	20 (38.5)	13 (25.0)	
	6	22 (42.3)	26 (50.0)	
	Others	4 (7.7)	7 (13.5)	
R-primary tumor site	Upper outer quadrant	20 (38.5)	32 (61.5)	0.265
	Lower outer quadrant	2 (3.8)	1 (1.9)	
	Lower inner quadrant	3 (5.8)	1 (1.9)	
	Upper inner quadrant	7 (13.5)	4 (7.7)	
	Central	3 (5.8)	1 (1.9)	
	Unknown	17 (32.7)	13 (25.0)	
R-LNM	No	38 (73.1)	29 (55.8)	0.101
	Yes	14 (26.9)	23 (44.2)	
R-BIRADS	4	3 (5.8)	0 (0.0)	0.238
	5	9 (17.3)	11 (21.2)	
	6	23 (44.2)	28 (53.8)	
	Others	17 (32.7)	13 (25.0)	
Clinical T stage	T1	8 (15.4)	7 (13.5)	0.946
	T2	29 (55.8)	28 (53.8)	
	T3	6 (11.5)	8 (15.4)	
	T4	9 (17.3)	9 (17.3)	
Clinical N stage	N0	11 (21.2)	5 (9.6)	0.240
	N1	15 (28.8)	20 (38.5)	
	N2	20 (38.5)	17 (32.7)	
	N3	6 (11.5)	10 (19.2)	
Clinical TNM stage	I	2 (3.8)	1 (1.9)	0.663
	II	21 (40.4)	18 (34.6)	
	III	29 (55.8)	33 (63.5)	
Type of surgery	Mastectomy	48 (92.3)	40 (76.9)	0.057
	Breast-conserving surgery	4 (7.7)	12 (23.1)	
P-tumor size	≤2cm	25 (48.1)	20 (38.5)	0.383
	>2 and <5cm	25 (48.1)	27 (51.9)	
	≥5cm	2 (3.8)	5 (9.6)	
Histologic grade	I	2 (3.8)	4 (7.7)	0.458
	II	31 (59.6)	34 (65.4)	
	III	19 (36.5)	14 (26.9)	
Pathological T stage	Tis/T0	4 (7.7)	0 (0.0)	0.196
	T1	21 (40.4)	20 (38.5)	
	T2	24 (46.2)	25 (48.1)	
	T3	1 (1.9)	1 (1.9)	
	T4	2 (3.8)	6 (11.5)	
Pathological N stage	N0	19 (36.5)	12 (23.1)	0.489
	N1	12 (23.1)	15 (28.8)	
	N2	8 (15.4)	11 (21.2)	
	N3	13 (25.0)	14 (26.9)	
Pathological TNM stage	Tis/T0	2 (3.8)	0 (0.0)	0.171
	I	11 (21.2)	5 (9.6)	
	II	17 (32.7)	21 (40.4)	
	III	22 (42.3)	26 (50.0)	
Total lymph node	<24	25 (48.1)	28 (53.8)	0.695
	≥24	27 (51.9)	24 (46.2)	
Positive lymph node	<2	24 (46.2)	19 (36.5)	0.426
	≥2	28 (53.8)	33 (63.5)	
Lymph vessel invasion	Negative	28 (53.8)	34 (65.4)	0.318
	Positive	24 (46.2)	18 (34.6)	
Neural invasion	Negative	40 (76.9)	41 (78.8)	1.000
	Positive	12 (23.1)	11 (21.2)	
Postoperative complications	No	47 (90.4)	51 (98.1)	0.207
	Yes	5 (9.6)	1 (1.9)	
Postoperative chemotherapy	No	18 (34.6)	12 (23.1)	0.279
	Yes	34 (65.4)	40 (76.9)	
Postoperative radiotherapy	No	15 (28.8)	10 (19.2)	0.359
	Yes	37 (71.2)	42 (80.8)	
Postoperative endocrine therapy	No	21 (40.4)	23 (44.2)	0.843
	Yes	31 (59.6)	29 (55.8)	
Postoperative targeted therapy	No	34 (65.4)	38 (73.1)	0.524
	Yes	18 (34.6)	14 (26.9)	

#Abbreviations: BMI, Body mass index; US, Ultrasonic sound; LNM, Lymph node metastasis; TNM; Tumor node metastasis; M, Mammography; BIRADS, Breast Imaging Reporting and Data System.

**Table 2:** Common hematologic index and oxidative stress indicators

	Level	Low BCIOSS	High BCIOSS	p
n		52	52	
ALT	<17	23 (44.2)	29 (55.8)	0.327
	≥17	29 (55.8)	23 (44.2)	
AST	<17	24 (46.2)	22 (42.3)	0.843
	≥17	28 (53.8)	30 (57.7)	
LDH	<166	26 (50.0)	26 (50.0)	1.000
	≥166	26 (50.0)	26 (50.0)	
GGT	<17	24 (46.2)	24 (46.2)	1.000
	≥17	28 (53.8)	28 (53.8)	
ALP	<65	24 (46.2)	25 (48.1)	1.000
	≥65	28 (53.8)	27 (51.9)	
ALB	<45.0	41 (78.8)	12 (23.1)	<0.001
	≥45.0	11 (21.2)	40 (76.9)	
CRP	<0.35	21 (40.4)	30 (57.7)	0.117
	≥0.35	31 (59.6)	22 (42.3)	
BUN	<4.3	21 (40.4)	30 (57.7)	0.117
	≥4.3	31 (59.6)	22 (42.3)	
CRE	<56.60	26 (50.0)	27 (51.9)	1.000
	≥56.60	26 (50.0)	25 (48.1)	
URIC	<253	21 (40.4)	29 (55.8)	0.169
	≥253	31 (59.6)	23 (44.2)	
TBA	<2.5	26 (50.0)	24 (46.2)	0.844
	≥2.5	26 (50.0)	28 (53.8)	
TBIL	<8.81	21 (40.4)	32 (61.5)	0.050
	≥8.81	31 (59.6)	20 (38.5)	
DBIL	<3	18 (34.6)	33 (63.5)	0.006
	≥3	34 (65.4)	19 (36.5)	
IBIL	<5.9	21 (40.4)	32 (61.5)	0.050
	≥5.9	31 (59.6)	20 (38.5)	
TP	<70.6	30 (57.7)	21 (40.4)	0.117
	≥70.6	22 (42.3)	31 (59.6)	
G	<25.80	27 (51.9)	24 (46.2)	0.695
	≥25.80	25 (48.1)	28 (53.8)	
A/G	<1.72	28 (53.8)	19 (36.5)	0.115
	≥1.72	24 (46.2)	33 (63.5)	
Lpa	<22.50	27 (51.9)	25 (48.1)	0.845
	≥22.50	25 (48.1)	27 (51.9)	
SOD	<164.10	31 (59.6)	21 (40.4)	0.078
	≥164.10	21 (40.4)	31 (59.6)	
HCY	<10.80	19 (36.5)	30 (57.7)	0.049
	≥10.80	33 (63.5)	22 (42.3)	
PALB	<23.80	28 (53.8)	22 (42.3)	0.326
	≥23.80	24 (46.2)	30 (57.7)	
CA125	<14.85	29 (55.8)	24 (46.2)	0.433
	≥14.85	23 (44.2)	28 (53.8)	
CA153	<12.70	27 (51.9)	26 (50.0)	1.000
	≥12.70	25 (48.1)	26 (50.0)	
CEA	<1.81	23 (44.2)	30 (57.7)	0.239
	≥1.81	29 (55.8)	22 (42.3)	
DD	<0.30	26 (50.0)	19 (36.5)	0.235
	≥0.30	26 (50.0)	33 (63.5)	
FIB	<2.87	29 (55.8)	24 (46.2)	0.433
	≥2.87	23 (44.2)	28 (53.8)	
INR	<0.93	20 (38.5)	27 (51.9)	0.237
	≥0.93	32 (61.5)	25 (48.1)	
FDP	<1.50	19 (36.5)	28 (53.8)	0.115
	≥1.50	33 (63.5)	24 (46.2)	
W	<5.92	29 (55.8)	22 (42.3)	0.239
	≥5.92	23 (44.2)	30 (57.7)	
R	<4.36	34 (65.4)	17 (32.7)	0.002
	≥4.36	18 (34.6)	35 (67.3)	
Hb	<130	30 (57.7)	20 (38.5)	0.077
	≥130	22 (42.3)	32 (61.5)	
N	<3.66	29 (55.8)	22 (42.3)	0.239
	≥3.66	23 (44.2)	30 (57.7)	
L	<1.75	23 (44.2)	28 (53.8)	0.433
	≥1.75	29 (55.8)	24 (46.2)	
M	<0.37	27 (51.9)	23 (44.2)	0.556
	≥0.37	25 (48.1)	29 (55.8)	
E	<0.06	17 (32.7)	28 (53.8)	0.048
	≥0.06	35 (67.3)	24 (46.2)	
B	<0.02	12 (23.1)	14 (26.9)	0.821
	≥0.02	40 (76.9)	38 (73.1)	
P	<234	28 (53.8)	22 (42.3)	0.326
	≥234	24 (46.2)	30 (57.7)	

#Abbreviations: ALT, Alanine aminotransferase; AST, Aspartate aminotransferase; LDH, Lactate dehydrogenase; GGT, γ-glutamyl transpeptidase; ALP, Alkaline phosphatase; ALB, Albumin; CRP, C-reactive protein; BUN, Blood urea nitrogen; CRE, Creatinine; URIC, Uric acid; TBA, Total bile acids; TBIL, Total bilirubin; DBIL, Direct bilirubin; IBIL, Indirect bilirubin; TP, Total protein; G, Globularproteins; A/G, Albumin/Globularproteins; Lpa, Lipoprotein; SOD, Superoxide dismutase; HCY, Homocysteine; PALB, Prealbumin; CA125, Cancer antigen 125; CA153, Cancer antigen 153; CEA, Carcinoembryonic antigen; DD, D-Dimer; FIB, Fibrinogen; INR, international normalized ratio; FDP, Fibrinogen degradation products; W, White blood cell; R, Red blood cell; Hb, Hemoglobin; N, Neutrophils; L, Lymphocyte; M, Monocyte; E, eosinophil; B, Basophil; P, Platelet.

**Table 3:** Association between BCIOSS and chemotherapy

	Level	Low BCIOSS	High BCIOSS	p
n		52	52	
Neoadjuvant chemotherapy regimen	AC/ACF	3 (5.8)	1 (1.9)	0.636
	CT/ACT	4 (7.7)	6 (11.5)	
	AT	24 (46.2)	29 (55.8)	
	TP	12 (23.1)	9 (17.3)	
	Others	9 (17.3)	7 (13.5)	
Neoadjuvant chemotherapy times	<6	18 (34.6)	16 (30.8)	0.834
	≥6	34 (65.4)	36 (69.2)	
Response	PR	29 (55.8)	31 (59.6)	0.580
	SD	22 (42.3)	21 (40.4)	
	PD	1 (1.9)	0 (0.0)	
Decreased appetite	No	9 (17.3)	8 (15.4)	1.000
	Yes	43 (82.7)	44 (84.6)	
Nausea	No	5 (9.6)	6 (11.5)	1.000
	Yes	47 (90.4)	46 (88.5)	
Vomiting	No	28 (53.8)	22 (42.3)	0.326
	Yes	24 (46.2)	30 (57.7)	
Diarrhea	No	49 (94.2)	48 (92.3)	1.000
	Yes	3 (5.8)	4 (7.7)	
Mouth ulcers	No	52 (100.0)	50 (96.2)	0.475
	Yes	0 (0.0)	2 (3.8)	
Alopecia	No	27 (51.9)	21 (40.4)	0.325
	Yes	25 (48.1)	31 (59.6)	
Peripheral neurotoxicity	No	45 (86.5)	42 (80.8)	0.596
	Yes	7 (13.5)	10 (19.2)	
Anemia	Grade 0	26 (50.0)	29 (55.8)	0.694
	Grade 1-2	26 (50.0)	23 (44.2)	
Leukopenia	Grade 0	12 (23.1)	12 (23.1)	0.483
	Grade 1-2	25 (48.1)	30 (57.7)	
	Grade 3-4	15 (28.8)	10 (19.2)	
Neutropenia	Grade 0	9 (17.3)	11 (21.2)	0.801
	Grade 1-2	22 (42.3)	19 (36.5)	
	Grade 3-4	21 (40.4)	22 (42.3)	
Thrombocytopenia	Grade 0	37 (71.2)	42 (80.8)	0.359
	Grade 1-2	15 (28.8)	10 (19.2)	
Gastrointestinal reaction	Grade 0	6 (11.5)	6 (11.5)	0.603
	Grade 1-2	45 (86.5)	46 (88.5)	
	Grade 3-4	1 (1.9)	0 (0.0)	
Myelosuppression	Grade 0	7 (13.5)	8 (15.4)	0.919
	Grade 1-2	15 (28.8)	16 (30.8)	
	Grade 3-4	30 (57.7)	28 (53.8)	
Hepatic dysfunction	Grade 0	33 (63.5)	33 (63.5)	1.000
	Grade 1-2	19 (36.5)	19 (36.5)	
Miller-Payne grade	1	3 (5.8)	6 (11.5)	0.082
	2	17 (32.7)	25 (48.1)	
	3	27 (51.9)	21 (40.4)	
	4	1 (1.9)	0 (0.0)	
	5	4 (7.7)	0 (0.0)	
Postoperative chemotherapy	No	18 (34.6)	12 (23.1)	0.279
	Yes	34 (65.4)	40 (76.9)	
Postoperative chemotherapy regimen	AC/ACF	4 (7.7)	5 (9.6)	0.715
	CT/ACT	2 (3.8)	4 (7.7)	
	AT	5 (9.6)	4 (7.7)	
	TP	9 (17.3)	8 (15.4)	
	Others	14 (26.9)	19 (36.5)	
	No	18 (34.6)	12 (23.1)	
Postoperative chemotherapy times	<4	26 (50.0)	22 (42.3)	0.555
	≥4	26 (50.0)	30 (57.7)	
Postoperative gastrointestinal reaction	Grade 0	24 (46.2)	17 (32.7)	0.107
	Grade 1-2	28 (53.8)	32 (61.5)	
	Grade 3-4	0 (0.0)	3 (5.8)	
Postoperative myelosuppression	Grade 0	24 (46.2)	19 (36.5)	0.466
	Grade 1-2	17 (32.7)	23 (44.2)	
	Grade 3-4	11 (21.2)	10 (19.2)	
Postoperative hepatic dysfunction	Grade 0	33 (63.5)	33 (63.5)	1.000
	Grade 1-2	19 (36.5)	19 (36.5)	

#Abbreviations: A, Anthracyclines; C, Cyclophosphamide; F, 5-Fluorouracil; T, Taxol; P, Platinum compounds.

**Table 4:** The relationship between BCIOSS and molecular pathology

	Level	Low BCIOSS	High BCIOSS	p
n		52	52	
Core needle biopsy				
Molecular subtype	Luminal A	5 (9.6)	3 (5.8)	0.651
	Luminal B HER2+	5 (9.6)	9 (17.3)	
	Luminal B HER2-	16 (30.8)	19 (36.5)	
	HER2 enriched	8 (15.4)	7 (13.5)	
	Triple negative	18 (34.6)	14 (26.9)	
ER	Negative	22 (42.3)	21 (40.4)	1.000
	Positive	30 (57.7)	31 (59.6)	
PR	Negative	22 (42.3)	20 (38.5)	0.842
	Positive	30 (57.7)	32 (61.5)	
HER2	Negative	39 (75.0)	37 (71.2)	0.825
	Positive	13 (25.0)	15 (28.8)	
Ki-67	Negative	9 (17.3)	11 (21.2)	0.804
	Positive	43 (82.7)	41 (78.8)	
Pathology after surgery				
Molecular subtype	Luminal A	7 (13.5)	10 (19.2)	0.619
	Luminal B HER2+	3 (5.8)	6 (11.5)	
	Luminal B HER2-	14 (26.9)	9 (17.3)	
	HER2 enriched	9 (17.3)	9 (17.3)	
	Triple negative	19 (36.5)	18 (34.6)	
ER	Negative	26 (50.0)	22 (42.3)	0.555
	Positive	26 (50.0)	30 (57.7)	
PR	Negative	25 (48.1)	25 (48.1)	1.000
	Positive	27 (51.9)	27 (51.9)	
HER2	Negative	40 (76.9)	40 (76.9)	1.000
	Positive	12 (23.1)	12 (23.1)	
Ki-67	Negative	16 (30.8)	20 (38.5)	0.536
	Positive	36 (69.2)	32 (61.5)	
AR	Negative	46 (88.5)	45 (86.5)	1.000
	Positive	6 (11.5)	7 (13.5)	
CK5/6	Negative	37 (71.2)	38 (73.1)	1.000
	Positive	15 (28.8)	14 (26.9)	
E-cad	Negative	15 (28.8)	9 (17.3)	0.245
	Positive	37 (71.2)	43 (82.7)	
EGFR	Negative	30 (57.7)	27 (51.9)	0.694
	Positive	22 (42.3)	25 (48.1)	
P53	Negative	22 (42.3)	22 (42.3)	1.000
	Positive	30 (57.7)	30 (57.7)	
TOP2A	Negative	13 (25.0)	10 (19.2)	0.637
	Positive	39 (75.0)	42 (80.8)	

#Abbreviations: ER: Estrogen receptor, PR: Progesterone receptor; HER2: Human Epidermal Growth Factor Receptor 2; AR: Androgen receptor; E-cad: E-Cadherin; EGFR: Epidermal growth factor receptor; TOP2A: Topoisomerase II-α.

**Table 5:** Univariate and multivariate Cox proportional hazards regression model analysis of the potential factors associated with disease free survival (DFS) and overall survival (OS)

	DFS						OS					
		Univariate			Multivariate			Univariate			Multivariate	
Characteristics	HR	95%CI	P	HR	95%CI	P	HR	95%CI	P	HR	95%CI	P
BCIOSS (low vs. High)	0.326	0.138-0.767	0.010	0.163	0.045-0.596	0.006	0.284	0.121-0.670	0.004	0.168	0.056-0.500	0.001
Age (<46 vs. ≥46)	1.725	0.778-3.821	0.179				1.822	0.824-4.032	0.139			
BMI (<23.63 vs. ≥23.63)	1.203	0.569-2.544	0.629				1.256	0.594-2.656	0.550			
Family history (No vs. Yes)	0.960	0.408-2.260	0.926				1.057	0.449-2.486	0.900			
Menopause (No vs. Yes)	0.892	0.416-1.911	0.768				0.934	0.437-1.996	0.860			
ALT (<17 vs. ≥17)	1.770	0.816-3.838	0.148				2.005	0.925-4.347	0.078			
AST (<17 vs. ≥17)	1.972	0.868-4.479	0.105				2.114	0.928-4.815	0.075			
LDH (<166 vs. ≥166)	1.517	0.717-3.208	0.276				1.416	0.670-2.994	0.363			
GGT (<17 vs. ≥17)	1.717	0.776-3.798	0.182				1.952	0.881-4.323	0.099			
ALP (<65 vs. ≥65)	2.738	1.163-6.446	0.021	0.580	0.150-2.240	0.430	2.763	1.174-6.502	0.020	1.049	0.321-3.433	0.937
ALB (<45.0 vs. ≥45.0)	0.960	0.457-2.020	0.915				0.927	0.441-1.948	0.841			
CRP (<0.35 vs. ≥0.35)	0.927	0.441-1.949	0.841				0.850	0.403-1.793	0.670			
BUN (<4.3 vs. ≥4.3)	1.606	0.741-3.480	0.230				1.771	0.817-3.841	0.148			
CRE (<56.60 vs. ≥56.60)	0.945	0.450-1.985	0.881				1.029	0.490-2.159	0.940			
URIC (<253 vs. ≥253)	0.968	0.460-2.037	0.931				1.050	0.498-2.213	0.898			
TBA (<2.5 vs. ≥2.5)	2.735	1.230-6.083	0.014	5.607	1.783-17.635	0.003	2.538	1.143-5.636	0.022	2.861	0.927-8.828	0.067
TBIL (<8.81 vs. ≥8.81)	0.567	0.262-1.230	0.151				0.551	0.253-1.198	0.132			
DBIL (<3 vs. ≥3)	0.874	0.414-1.846	0.725				0.909	0.430-1.922	0.803			
IBIL (<5.9 vs. ≥5.9)	0.545	0.251-1.183	0.125				0.550	0.253-1.196	0.131			
TP (<70.6 vs. ≥70.6)	1.933	0.892-4.190	0.095				1.870	0.862-4.058	0.113			
G (<25.80 vs. ≥25.80)	1.747	0.817-3.734	0.150				1.550	0.722-3.327	0.261			
A/G (<1.72 vs. ≥1.72)	0.360	0.166-0.782	0.010	0.977	0.264-3.608	0.972	0.406	0.187-0.884	0.023	0.881	0.273-2.842	0.832
Lpa (<22.50 vs. ≥22.50)	1.274	0.606-2.680	0.523				0.964	0.459-2.024	0.923			
SOD (<164.10 vs. ≥164.10)	0.263	0.112-0.620	0.002	1.573	0.402-6.161	0.515	0.285	0.121-0.673	0.004	1.033	0.318-3.352	0.957
HCY (<164.10 vs. ≥164.10)	0.982	0.467-2.065	0.962				1.001	0.476-2.109	0.997			
PALB (<23.80 vs. ≥23.80)	2.199	0.993-4.871	0.052				2.031	0.919-4.492	0.080			
CA125 (<14.85 vs. ≥14.85)	0.847	0.400-1.794	0.665				0.760	0.359-1.609	0.474			
CA153 (<12.70 vs. ≥12.70)	2.772	1.220-6.301	0.015	5.149	1.442-18.381	0.012	2.975	1.300-6.808	0.010	1.829	0.566-5.909	0.313
CEA (<1.81 vs. ≥1.81)	2.596	1.143-5.897	0.023	0.990	0.312-3.145	0.986	3.022	1.329-6.870	0.008	1.937	0.673-5.578	0.221
ABO blood type (A+B+O vs. AB)	1.089	0.758-1.566	0.643				1.084	0.749-1.570	0.667			
W (<5.92 vs. ≥5.92)	0.627	0.296-1.328	0.223				0.700	0.329-1.490	0.355			
R (<4.36 vs. ≥4.36)	0.614	0.288-1.312	0.208				0.536	0.250-1.147	0.108			
Hb (<130 vs. ≥130)	0.817	0.389-1.717	0.594				0.785	0.372-1.657	0.526			
N (<3.66 vs. ≥3.66)	0.608	0.287-1.289	0.195				0.677	0.318-1.440	0.311			
L (<1.75 vs. ≥1.75)	0.814	0.387-1.714	0.588				0.863	0.409-1.821	0.700			
M (<0.37 vs. ≥0.37)	1.149	0.547-2.416	0.714				0.978	0.466-2.053	0.952			
E (<0.06 vs. ≥0.06)	2.507	1.065-5.903	0.035	1.836	0.631-5.342	0.265	2.352	0.999-5.537	0.050			
B (<0.02 vs. ≥0.02)	0.830	0.365-1.887	0.657				0.800	0.352-1.820	0.594			
P (<234 vs. ≥234)	1.603	0.749-3.429	0.224				1.561	0.731-3.334	0.250			
Primary tumor site (Upper outer quadrant vs. Others)	0.813	0.591-1.118	0.202				0.835	0.607-1.150	0.270			
US-LNM (No vs. Yes)	2.777	1.314-5.866	0.007	3.625	1.079-12.173	0.037	2.577	1.207-5.500	0.014	1.878	0.641-5.506	0.251
US-BIRADS (4+5 vs. 6)	1.572	0.842-2.933	0.155				1.680	0.882-3.199	0.114			
Clinical T stage (T1 vs. T2+ T3+T4)	1.594	1.098-2.313	0.014	1.387	0.693-2.779	0.356	1.591	1.090-2.321	0.016	1.376	0.698-2.710	0.356
Clinical N stage (N0 vs. N1+N2+N3)	1.317	0.876-1.981	0.186				1.232	0.827-1.835	0.305			
Clinical TNM stage (I vs. II+III)	1.676	0.810-3.465	0.164				1.486	0.726-3.040	0.279			
Type of surgery (Mastectomy vs. Breast-conserving surgery)	0.386	0.092-1.626	0.194				0.359	0.085-1.515	0.163			
Pathological tumor size (≤2cm vs. >2cm)	2.237	1.194-4.193	0.012	2.752	1.008-7.509	0.048	2.147	1.131-4.076	0.020	1.585	0.622-4.042	0.335
Miller-Payne grade (MPG) (1+2+3 vs. 4+5)	1.079	0.682-1.709	0.744				1.134	0.710-1.809	0.599			
Histologic.grade (I vs. II+III)	1.782	0.900-3.528	0.097				1.474	0.749-2.901	0.262			
Pathological T stage (T1 vs. T2+ T3+T4)	1.611	1.122-2.312	0.010	0.557	0.250-1.242	0.153	1.777	1.204-2.622	0.004	1.380	0.714-2.667	0.338
Pathological N stage (N0 vs. N1+N2+N3)	1.275	0.915-1.776	0.151				1.275	0.920-1.767	0.144			
Pathological TNM stage (Tis/T0+I vs. II+III)	1.487	0.887-2.495	0.132				1.518	0.881-2.618	0.133			
TLN (<24 vs. ≥24)	0.600	0.276-1.300	0.195				0.532	0.245-1.153	0.110			
PLN (<2 vs. ≥2)	1.111	0.520-2.375	0.786				1.226	0.573-2.624	0.600			
Molecular subtype (Luminal A+Luminal B HER2+/ HER2- vs. HER2 enriched + Triple negative)	1.246	0.946-1.640	0.118				1.175	0.900-1.535	0.235			
ER (Negative vs. Positive)	0.837	0.398-1.762	0.640				0.977	0.464-2.058	0.952			
PR (Negative vs. Positive)	0.914	0.434-1.925	0.813				1.106	0.525-2.328	0.791			
HER2 (Negative vs. Positive)	1.218	0.514-2.886	0.655				1.270	0.539-2.995	0.585			
Ki-67 (Negative vs. Positive)	2.395	0.970-5.914	0.058				2.094	0.849-5.167	0.109			
AR (Negative vs. Positive)	1.164	0.403-3.361	0.778				1.192	0.412-3.453	0.746			
CK5/6 (Negative vs. Positive)	1.306	0.574-2.968	0.524				1.007	0.443-2.292	0.986			
E-cad (Negative vs. Positive)	1.234	0.519-2.931	0.634				1.029	0.434-2.441	0.948			
EGFR (Negative vs. Positive)	1.317	0.626-2.770	0.469				1.095	0.521-2.302	0.811			
P53 (Negative vs. Positive)	1.605	0.739-3.486	0.231				1.564	0.721-3.393	0.257			
TOP2A (Negative vs. Positive)	0.647	0.293-1.432	0.283				0.618	0.279-1.368	0.235			
Lymph vessel invasion (No vs. Yes)	1.947	0.924-4.105	0.080				2.050	0.974-4.315	0.059			
Neural invasion (No vs. Yes)	2.232	1.028-4.848	0.042	3.054	0.801-11.640	0.102	2.170	1.000-4.706	0.050			
Postoperative chemotherapy (No vs. Yes)	0.963	0.409-2.268	0.930				1.073	0.453-2.539	0.873			
Postoperative radiotherapy (No vs. Yes)	0.382	0.179-0.817	0.013	1.265	0.429-3.732	0.670	0.449	0.210-0.958	0.038	1.193	0.397-3.582	0.753
Postoperative endocrine therapy (No vs. Yes)	0.375	0.177-0.795	0.011	0.166	0.051-0.543	0.003	0.442	0.209-0.935	0.033	0.226	0.074-0.695	0.009
Postoperative targeted therapy (No vs. Yes)	2.374	1.117-5.045	0.025	1.776	0.560-5.638	0.330	2.388	1.132-5.036	0.022	2.440	0.809-7.360	0.113

#Abbreviations: BMI, Body mass index; US, Ultrasonic sound; TNM; Tumor node metastasis; BIRADS, Breast Imaging Reporting and Data System; ALT, Alanine aminotransferase; AST, Aspartate aminotransferase; LDH, Lactate dehydrogenase; GGT, γ-glutamyl transpeptidase; ALP, Alkaline phosphatase; ALB, Albumin; CRP, C-reactive protein; BUN, Blood urea nitrogen; CRE, Creatinine; URIC, Uric acid; TBA, Total bile acids; TBIL, Total bilirubin; DBIL, Direct bilirubin; IBIL, Indirect bilirubin; TP, Total protein; G, Globularproteins; A/G, Albumin/Globularproteins; Lpa, Lipoprotein; SOD, Superoxide dismutase; HCY, Homocysteine; PALB, Prealbumin; CA125, Cancer antigen 125; CA153, Cancer antigen 153; CEA, Carcinoembryonic antigen; DD, D-Dimer; FIB, Fibrinogen; INR, international normalized ratio; FDP, Fibrinogen degradation products; W, White blood cell; R, Red blood cell; Hb, Hemoglobin; N, Neutrophils; L, Lymphocyte; M, Monocyte; E, eosinophil; B, Basophil; P, Platelet; MPG: Miller-Payne grade; TLN: Total lymph node; PLN: Positive lymph node; ER: Estrogen receptor, PR: Progesterone receptor; HER2: Human Epidermal Growth Factor Receptor 2; AR: Androgen receptor; E-cad: E-Cadherin; EGFR: Epidermal growth factor receptor; TOP2A: Topoisomerase II-α.
